# Relationship between muscle mass and fraction of intramuscular adipose tissue of the quadriceps in older inpatients

**DOI:** 10.1371/journal.pone.0263973

**Published:** 2022-02-17

**Authors:** Naoki Akazawa, Masaki Kishi, Toshikazu Hino, Ryota Tsuji, Kimiyuki Tamura, Akemi Hioka, Hideki Moriyama

**Affiliations:** 1 Department of Physical Therapy, Faculty of Health and Welfare, Tokushima Bunri University, Tokushima, Tokushima, Japan; 2 Department of Rehabilitation, Kasei Tamura Hospital, Wakayama, Wakayama, Japan; 3 Life and Medical Sciences Area, Health Sciences Discipline, Kobe University, Kobe, Hyogo, Japan; University of Houston, UNITED STATES

## Abstract

**Background:**

In 2021, the International Society of Physical and Rehabilitation Medicine (ISPRM) special interest group on sarcopenia included the quadriceps thickness assessed with ultrasound image as an indicator of muscle mass in the diagnosis criteria of sarcopenia. If quadriceps echo intensity of older inpatients is to be a strong predictor of quadriceps thickness, muscle quality of the quadriceps may be estimated by the muscle mass when diagnosing sarcopenia using the criteria of ISPRM.

**Objective:**

This study aimed to examine the association between muscle mass and fraction of intramuscular adipose tissue of the quadriceps in older inpatients.

**Methods:**

This cross-sectional study included 399 inpatients aged ≥ 65 years. Primary outcomes were muscle mass and fraction of intramuscular adipose tissue of the quadriceps. Images were acquired using a B-mode ultrasound. Muscle mass and fraction of intramuscular adipose tissue of the quadriceps were assessed based on the muscle thickness and echo intensity, respectively. A multiple regression analysis (forced entry method) was performed to confirm whether quadriceps echo intensity was related to quadriceps thickness even after adjusting for other factors.

**Results:**

In the multiple regression analyses for both male and female models, quadriceps echo intensity (male: β = − 0.537, p < 0.001; female: β = − 0.438, p < 0.001), Geriatric Nutritional Risk Index (male: β = 0.236, p < 0.001; female: β = 0.213, p < 0.001), and subcutaneous fat thickness of the thigh (male: β = 0.197, p < 0.001; female: β = 0.248, p < 0.001) were independently and significantly associated with quadriceps thickness.

**Conclusions:**

Our results show that there is a negative and significant association between muscle mass and fraction of intramuscular adipose tissue in older inpatients. Muscle quality of the quadriceps in older inpatients may be estimated to some extent by the muscle mass.

## Introduction

Sarcopenia is widely known to lead to a decline in the activities of daily living [1], an increase in the fall risk [2], and an increase in mortality [1]. Therefore, sarcopenia has been recognized as a serious problem among older persons [1, 2].

In 2021, the International Society of Physical and Rehabilitation Medicine (ISPRM) special interest group on sarcopenia included the quadriceps thickness assessed with ultrasound image as an indicator of muscle mass in the diagnosis criteria of sarcopenia [3]. The diagnosis criteria of sarcopenia in ISPRM will be considered to be widely spread because muscle mass measurement using ultrasound is easy, inexpensive, non-invasive, and requires less time to perform. Furthermore, considering the installation situation of ultrasound device, the diagnosis criteria of sarcopenia in ISPRM may be more used in clinical settings than in community settings.

On the other hand, the European Working Group of Sarcopenia in Older People2 (EWGSOP2) [4] suggested the importance of assessing not only muscle mass but also muscle quality, which includes the infiltration of fat into muscle, ratio of muscle strength to appendicular skeletal muscle mass, and bioimpedance analysis-derived phase angle, in the diagnosis of sarcopenia. Among the muscle quality parameters, intramuscular adipose tissue has been shown to be more closely related to muscle strength [5, 6], stand-up, sit-down [5, 7], and gait abilities [6, 8, 9], activities of daily living [10, 11], swallowing function [12, 13], onset of hip fracture [14], and mortality [15] than to muscle mass. Fraction of intramuscular adipose tissue is assessed with the echo intensity of the ultrasound image [5–13]. However, setting the reference value for echo intensity is difficult because measurement conditions (e.g., differences of ultrasound device, probe, and scanning conditions) influence echo intensity [16, 17]. In fact, the EWGSOP2 has not provided the cut-off value of echo intensity in the diagnosis criteria of sarcopenia [4].

A previous study [18] reported that quadriceps thickness assessed with ultrasound image is closely related to quadriceps echo intensity in community-dwelling older persons (male: r = − 0.734, p < 0.01; female: r = − 0.565, p < 0.01). However, this detailed relationship has not yet been assessed in older inpatients who experience various conditions, including nutritional deficiencies, inflammatory conditions, changes in the activities of daily living, and swallowing problems, which affect their muscle mass and intramuscular adipose tissue [8, 10–13, 19–21].

If quadriceps echo intensity of older inpatients is to be a strong predictor of quadriceps thickness, muscle quality of the quadriceps may be estimated by the muscle mass. In other words, when assessing quadriceps thickness in older inpatients in accordance with the diagnosis criteria of sarcopenia in ISPRM [3], muscle quality may be estimated to some extent. Examining whether the fraction of intramuscular adipose tissue of the quadriceps is related to the muscle thickness in older inpatients is important for deeply understanding the value which is indicated by muscle thickness of the quadriceps. This study aimed to examine the association between muscle mass and fraction of intramuscular adipose tissue of the quadriceps in older inpatients.

## Materials and methods

### Study design and participants

This cross-sectional study included older inpatients (aged ≥ 65 years) who were referred to the Department of Rehabilitation at X Hospital. This hospital had subacute and convalescent rehabilitation wards. Patients who had undergone thigh amputation and were therefore unable to undergo the assessment of the muscle mass and intramuscular adipose tissue of the quadriceps were excluded from the study. In total, 449 inpatients were recruited between June 2017 and March 2020. Of these, 50 patients were excluded because they were aged < 65 years (n = 35), lacked necessary data (n = 14), or had undergone thigh amputation (n = 1). Consequently, 399 inpatients participated in the study. All the participants underwent a rehabilitation that was conducted by a physical, occupational, or speech therapist for 40 to 60 min per day, 5 to 6 days per week. The study protocol was approved by the ethics committee of our institution, and written informed consent was obtained from all the participants before participation in the study.

### Outcome measures

Primary outcomes were muscle mass and intramuscular adipose tissue of the quadriceps. We also measured other characteristics, including disease type, age, sex, height, weight, body mass index (BMI), length of hospital stay, subcutaneous fat mass of the thigh, swallowing function, inflammation, nutritional status, activity status, comorbidities, and number of medications. Most older inpatients at our hospital were admitted from another acute-phase hospital. For these patients, the length of hospital stay was measured as the total length of stay in both hospitals.

### Measurements of the muscle mass and fraction of intramuscular adipose tissue of the quadriceps and subcutaneous fat mass of the thigh

Transverse ultrasound images were acquired using a B-mode ultrasound device (Nanomaxx; SonoSite Japan, Tokyo, Japan) with a linear-array probe (L25n/13–6 MHz; Nanomaxx; SonoSite Japan). Muscle mass and fraction of intramuscular adipose tissue of the rectus femoris and vastus intermedius were assessed on the basis of the muscle thickness and echo intensity [5–13, 16–22], respectively. The validity of the muscle mass and fraction of intramuscular adipose tissue measurements using ultrasound was confirmed in recent studies using magnetic resonance imaging [23–25].

Images of the rectus femoris and vastus intermedius were obtained at 30% of their distance from the anterior superior iliac spine to the proximal end of the patella [6, 8, 10–13, 19–22]. The participants laid in the supine position with their lower limbs relaxed, while a water-soluble transmission gel was applied to the skin surface of their thighs. The probe was pressed perpendicularly and lightly against the skin to avoid deformation of the muscle. All the ultrasound images were recorded by the same investigator who had sufficient training in muscle thickness and echo intensity measurements. Muscle thickness of the rectus femoris was determined as the distance between the superficial adipose tissue-muscle interface and the deep muscle-muscle interface [6, 8, 10–13, 19–22], and muscle thickness of the vastus intermedius was determined as the distance between the superficial muscle-muscle interface and the bone-muscle interface [6, 8, 10–13, 19–22]. Echo intensity was measured in one region of interest in the rectus femoris and vastus intermedius, which was selected to include as much muscle as possible while avoiding the bone and surrounding fascia [5–13, 19–22]. To standardize all the echo intensity measurements, the gain status was uniform with the initial setting of the ultrasound system. In addition, the image depth was uniform at 60 mm in all the muscle thickness and echo intensity measurements. Muscle thickness and echo intensity were determined using ImageJ 1.49 software (National Institutes of Health, Bethesda, MD, USA) [5–13, 19–22]. Echo intensity was determined by computer-assisted 8-bit gray-scale analysis, and the mean echo intensities of the regions of interest were expressed as values from 0 (black) to 255 (white) [5–13, 16–22]. A higher echo intensity indicates a greater fraction of intramuscular adipose tissue [26].

The sum of the thicknesses of the rectus femoris and vastus intermedius was used as a measure of quadriceps thickness [6, 8, 10–13, 19–22]. The mean thicknesses of the right and left quadriceps were used for the analysis [6, 8, 10–13, 19–22]. The echo intensity of the quadriceps was calculated as the mean echo intensities of the rectus femoris and vastus intermedius [6, 8, 10–13, 19–22]. The mean echo intensities of the right and left quadriceps were used for analysis [6, 8, 10–13, 19–22]. The methods used in measuring the muscle thickness and echo intensity of the rectus femoris and vastus intermedius in our study group reportedly had a high reliability (intraclass correlation coefficients [1.1] = 0.857–0.959) [22]. In addition, the subcutaneous fat mass of the thigh was assessed based on the subcutaneous fat thickness. Subcutaneous fat thickness was determined as the distance between the dermis and adipose tissue interface and the muscle-adipose tissue interface [6, 8, 10–13, 19–22]. The mean values of the right and left subcutaneous fat thicknesses of the thighs were used for the analysis [6, 8, 10–13, 19–22].

### Measures of other characteristics

Swallowing functions were assessed using the Food Intake Level Scale (FILS) [27], which is a 10-point observer-rated scale. Levels 1 to 3 indicate various degrees of non-oral feeding; levels 4 to 6 relate to various degrees of oral food intake and alternative nutrition, such as enteral and parenteral nutrition; levels 7 to 9 refer to various degrees of oral intake alone; and level 10 indicates normal oral food intake. Inflammatory status was assessed using the C-reactive protein (CRP) concentration. Nutritional status was assessed using the Geriatric Nutritional Risk Index (GNRI) [28]. The GNRI was calculated using the following formula: GNRI = (14.89 × serum albumin [g/dl]) + (41.7 × weight [kg]/ideal body weight) [28]. Ideal body weight was defined as a BMI of 22.0 kg/m^2^ [29]. A previous study [29] of Japanese patients revealed a high correlation coefficient between the GNRI calculated with an ideal BMI of 22.0 kg/m^2^ and that calculated by the Lorentz formula (r = 0.99). If weight/ideal body weight was ≥ 1.0, the ratio was set to 1 [28]. Comorbidities were evaluated using the updated Charlson comorbidity index (UCCI) [30]. Activities of daily living were assessed using the Functional Independence Measure (FIM) [31]. This assessment consists of 13 motor and 5 cognitive items. Motor-FIM items were used in the present study, which included eating; grooming; bathing; dressing the upper and lower body; toileting; bladder and bowel management; bed, chair, wheelchair, toilet, or tub transfer; walk/wheelchair; and the use of stairs. Each item was scored from 1 to 7 based on the amount of assistance required. The motor-FIM score ranged from 13 to 91, with a higher score indicating a higher ability to perform the activities of daily living.

### Statistical analysis

All the statistical analyses were conducted using SPSS version 24 software (IBM SPSS Japan, Tokyo, Japan). The results of the Shapiro-Wilk test and the normality distributions of all the variables were not confirmed. Therefore, differences in the characteristics of the male and female inpatients were assessed using the Mann-Whitney U test, and the associations between muscle thickness and echo intensity of the quadriceps in male and female inpatients were assessed using the Kendall’s tau rank correlation coefficient.

We used a simple regression analysis to assess the contribution rate of quadriceps echo intensity to quadriceps thickness in each sex. Multiple regression analyses (forced entry and stepwise methods) were performed to confirm whether quadriceps echo intensity was related to quadriceps thickness, even after adjusting for other variables in each gender. The independent variables were quadriceps echo intensity, age, length of hospital stay, motor-FIM score, FILS, GNRI, CRP, UCCI, number of medications, and subcutaneous fat thickness of the thigh. Previous studies [8, 10–13, 19–21, 32, 33] reported that muscle mass and intramuscular adipose tissue of the quadriceps or thigh are affected by the length of hospital stay, activities of daily living, swallowing function, nutritional and inflammatory statuses, and subcutaneous fat thickness of the thigh. Therefore, we selected length of hospital stay, motor-FIM score, FILS, GNRI, CRP, and subcutaneous fat thickness of the thigh as confounding factors in addition to the basic characteristics. A p-value < 0.05 was considered to indicate statistical significance. In addition, we calculated the effect size (f^2^) of the multiple regression analysis using the following equation: R^2^/(1 − R^2^), and the statistical power of the analysis based on f^2^, an alpha error of 0.05, the total sample size, and the number of predictor variables. Statistical power was calculated using G* Power version 3.1.9.2 (Heinrich-Heine-Universität Düsseldorf, Düsseldorf, Germany).

## Results

**Fig 1:**
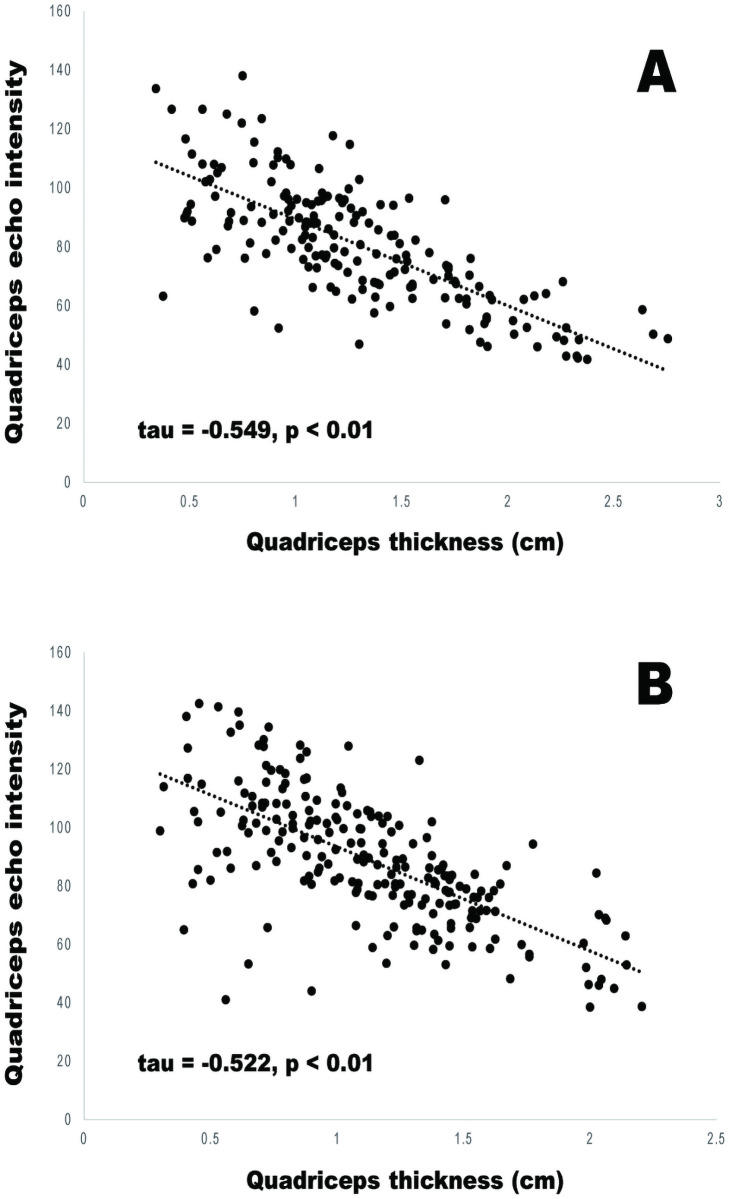
Association between muscle thickness and echo intensity of the quadriceps in male (A) and female (B) inpatients.

The number of male and female inpatients within the total study population (n = 399) was 180 and 219, respectively. Diseases found among the participants were stroke (n = 60), hip fractures (n = 46), compression fractures (n = 46), pubic fractures (n = 9), other fractures (n = 20), pneumonia (n = 63), heart disease (n = 27), spinal cord disease (n = 10), urinary tract infection (n = 11), and others (n = 107). Table 1 shows the characteristics of the participants. All the variables were expressed as median (interquartile range). Correlation analysis showed that there was a negative and significant association between quadriceps thickness and echo intensity in both genders (male: Kendall’s tau rank correlation coefficient = − 0.549, p < 0.01; female: Kendall’s tau rank correlation coefficient = − 0.522, p < 0.01). Fig 1 shows the association between muscle thickness and echo intensity of the quadriceps in male and female inpatients.

**Table 1:** Participants’ characteristics.

	Total	Male	Female	p-value*
	(n = 399)	(n = 180)	(n = 219)	
Age, years	83.0 (77.0–88.0)	82.0 (76.0–87.8)	84.0 (79.0–88.0)	0.004
Height, cm	152.0 (147.0–161.0)	162.0 (155.0–165.0)	148.0 (144.0–150.0)	< 0.001
Weight, kg	46.5 (39.5–54.0)	51.0 (44.9–57.0)	43.6 (36.2–49.6)	< 0.001
BMI, kg/m^2^	19.9 (17.4–22.5)	19.7 (17.5–22.3)	20.0 (17.4–22.6)	0.927
Length of hospital stay, days	25.0 (12.0–39.0)	25.5 (14.0–40.8)	25.0 (11.0–39.0)	0.481
Quadriceps thickness, cm	1.2 (0.9–1.5)	1.2 (0.9–1.6)	1.1 (0.8–1.4)	0.006
Quadriceps echo intensity (gray-scale range, 0–255)	84.1 (69.1–99.7)	79.7 (66.3–94.4)	87.0 (73.8–103.9)	< 0.001
Subcutaneous fat thickness of the thigh, cm	0.4 (0.3–0.5)	0.4 (0.2–0.5)	0.4 (0.3–0.6)	0.008
Food Intake Level Scale	8.0 (7.0–9.0)	8.0 (7.0–9.0)	8.0 (7.0–8.0)	0.435
C-reactive protein, mg/dl	0.5 (0.4–1.7)	0.6 (0.4–2.2)	0.4 (0.4–1.4)	0.101
Serum albumin, g/dl	3.4 (3.0–3.7)	3.3 (3.0–3.6)	3.4 (3.1–3.7)	0.265
Geriatric Nutritional Risk Index	87.6 (80.2–94.1)	87.6 (78.6–93.8)	87.6 (81.2–94.4)	0.563
Updated Charlson comorbidity index	2.0 (0.0–3.0)	2.0 (1.0–4.0)	2.0 (0.0–3.0)	< 0.001
Number of medications	7.0 (5.0–10.0)	7.0 (4.0–9.0)	7.0 (5.0–10.0)	0.307
Motor-FIM score	41.0 (21.0–62.0)	43.0 (20.3–63.0)	39.0 (21.0–60.0)	0.331

Data are presented as median (interquartile range).

BMI, body mass index; FIM, Functional Independence Measure.

*Mann-Whitney U test (male versus female).

Simple regression analysis showed that quadriceps echo intensity estimated the quadriceps thickness of male and female inpatients to be approximately 55% (β = − 0.739, p < 0.001, R^2^ = 0.546) and 46% (β = − 0.679, p < 0.001, R^2^ = 0.461), respectively. Results of the multiple regression analyses for male and female inpatients are shown in Tables 2–5. No multicollinearity was present among the independent variables in all regression models. In the multiple regression analysis for the male model with forced entry method, quadriceps echo intensity (β = − 0.537, p < 0.001), GNRI (β = 0.236, p < 0.001) and subcutaneous fat thickness of the thigh (β = 0.197, p < 0.001) were independently and significantly associated with quadriceps thickness (R^2^ = 0.654, f^2^ = 1.890, statistical power = 1.000) (Table 2). In the multiple regression analysis for the female model with forced entry method, quadriceps echo intensity (β = − 0.438, p < 0.001), GNRI (β = 0.213, p < 0.001), and subcutaneous fat thickness of the thigh (β = 0.248, p < 0.001) were independently and significantly associated with quadriceps thickness (R^2^ = 0.603, f^2^ = 1.518, statistical power = 1.000) (Table 3). Even in the multiple regression analyses with the stepwise method for the male and female models, quadriceps echo intensity, GNRI, and subcutaneous fat thickness of the thigh were independently and significantly associated with quadriceps thickness (male model [Table 4]: R^2^ = 0.635, f^2^ = 1.740, statistical power = 1.000; female model [Table 5]: R^2^ = 0.586, f^2^ = 1.415, statistical power = 1.000).

**Table 2:** Multiple regression analysis for muscle thickness in male model with forced entry method (n = 180).

	B	SE	95% Confidence interval of B	β	VIF	p-value
Quadriceps echo intensity	− 0.014	0.001	− 0.016, − 0.011	− 0.537	1.615	< 0.001
Age	0.000	0.004	− 0.007, 0.007	0.001	1.348	0.983
Length of hospital stay	− 0.002	0.001	− 0.003, 0.000	− 0.087	1.166	0.078
Subcutaneous fat thickness of the thigh	0.591	0.151	0.292, 0.889	0.197	1.249	< 0.001
Food Intake Level Scale	− 0.002	0.015	− 0.031, 0.027	− 0.007	1.624	0.902
C-reactive protein, mg/dl	0.009	0.007	− 0.006, 0.023	0.060	1.256	0.239
Geriatric Nutritional Risk Index	0.011	0.003	0.006, 0.017	0.236	1.774	< 0.001
Updated Charlson comorbidity index	0.006	0.010	− 0.015, 0.026	0.026	1.100	0.583
Number of medications	0.011	0.006	− 0.001, 0.023	0.087	1.116	0.070
Motor-FIM score	0.001	0.001	− 0.001, 0.004	0.052	1.639	0.375

B, partial regression coefficient; SE, standard error; β, standardized partial regression coefficient; VIF, variance inflation factor

**Table 3:** Multiple regression analysis for muscle thickness in female model with forced entry method (n = 219).

	B	SE	95% Confidence interval of B	β	VIF	p-value
Quadriceps echo intensity	− 0.008	0.001	− 0.010, − 0.006	− 0.438	1.508	< 0.001
Age	− 0.005	0.003	− 0.010, 0.001	− 0.081	1.256	0.099
Length of hospital stay	− 0.001	0.001	− 0.002, 0.001	− 0.055	1.212	0.256
Subcutaneous fat thickness of the thigh	0.401	0.085	0.233, 0.569	0.248	1.451	< 0.001
Food Intake Level Scale	− 0.002	0.013	− 0.029, 0.024	− 0.010	1.419	0.855
C-reactive protein, mg/dl	0.013	0.008	− 0.003, 0.030	0.076	1.130	0.101
Geriatric Nutritional Risk Index	0.008	0.002	0.004, 0.013	0.213	1.736	< 0.001
Updated Charlson comorbidity index	0.005	0.010	− 0.014, 0.024	0.023	1.141	0.622
Number of medications	0.000	0.005	− 0.010, 0.011	0.002	1.104	0.964
Motor-FIM score	0.001	0.001	− 0.001, 0.003	0.063	1.565	0.249

B, partial regression coefficient; SE, standard error; β, standardized partial regression coefficient; VIF, variance inflation factor

**Table 4:** Multiple regression analysis for muscle thickness in male model with stepwise method (n = 180).

	B	SE	95% Confidence interval of B	β	VIF	p-value
Quadriceps echo intensity	− 0.014	0.001	− 0.017, − 0.011	− 0.561	1.353	< 0.001
Geriatric Nutritional Risk Index	0.012	0.002	0.007, 0.017	0.248	1.255	< 0.001
Subcutaneous fat thickness of the thigh	0.554	0.149	0.261, 0.848	0.185	1.191	< 0.001

B, partial regression coefficient; SE, standard error; β, standardized partial regression coefficient; VIF, variance inflation factor

**Table 5:** Multiple regression analysis for muscle thickness in female model with stepwise method (n = 219).

	B	SE	95% Confidence interval of B	β	VIF	p-value
Quadriceps echo intensity	− 0.009	0.001	− 0.011, − 0.007	− 0.468	1.354	< 0.001
Subcutaneous fat thickness of the thigh	0.405	0.084	0.240, 0.569	0.250	1.384	< 0.001
Geriatric Nutritional Risk Index	0.009	0.002	0.005, 0.013	0.234	1.362	< 0.001

B, partial regression coefficient; SE, standard error; β, standardized partial regression coefficient; VIF, variance inflation factor

## Discussion

Our findings indicate that there is a negative and significant association between muscle mass and fraction of intramuscular adipose tissue of the quadriceps in older inpatients. Furthermore, this association was observed even after adjusting for age, activity, swallowing function, nutritional status, inflammation, medication status, comorbidities, subcutaneous fat mass, and length of hospital stay. Considering our results, muscle quality of the quadriceps in older inpatients is likely declining due to a decrease in muscle mass.

Quadriceps thickness has been shown to be closely related to quadriceps echo intensity in community-dwelling older persons [18]. Also in this study, a similar relationship between quadriceps thickness and echo intensity in older inpatients was observed. Furthermore, considering that muscle mass and intramuscular adipose tissue of the thigh assessed with computed tomography in older inpatients is similarly related to handgrip strength and motor function [34], the results of this study are considered to be valid. In addition, GNRI was independently and significantly related to quadriceps thickness in this study. Considering malnutrition is closely related to sarcopenia [35, 36], this result supported these previous results.

This study was the first to demonstrate that even among older inpatients with various nutritional, inflammation, and medication statuses, degrees of activity, swallowing abilities, and comorbidities, there is a negative association between muscle mass and fraction of intramuscular adipose tissue. A recent study [8] reported that compared to community-dwelling older persons matched for age, sex, and BMI, the muscle mass of the quadriceps in older inpatients is lower by 63% and intramuscular adipose tissue is higher approximately 1.7 times. In this way, although quantitative differences were observed in muscle mass and intramuscular adipose tissue among community-dwelling older persons and older inpatients, our results (i.e., there is a negative association between muscle mass and fraction of intramuscular adipose tissue) were similar to the results of a previous study [18] obtained from community-dwelling older persons. Considering our results, muscle quality of the quadriceps in older inpatients may be estimated to some extent by the muscle mass.

Although the EWGSOP2 [4] suggested the importance of assessing not only muscle mass but also muscle quality including intramuscular adipose tissue in the diagnosis of sarcopenia, setting the reference value for echo intensity is difficult because measurement conditions influence this value [16, 17]. More recently, the ISPRM special interest group on sarcopenia included the quadriceps thickness assessed with ultrasound image as an indicator of muscle mass in the diagnosis criteria of sarcopenia [3]. Considering there is no cut-off value of echo intensity for discriminating low muscle quality and muscle mass measurement using ultrasound is easy, inexpensive, non-invasive, and requires less time to perform, the diagnosis criteria of sarcopenia in ISPRM [3] will be considered to be widely spread. Furthermore, we consider that our results are contributed to deepening an understanding of quadriceps thickness value from the perspective of muscle quality. Based on the aforementioned circumstances and our findings, the muscle quality of the quadriceps will be possible to estimate from the result of muscle mass measurement when assessing quadriceps thickness in older inpatients in accordance with the diagnosis criteria of sarcopenia in ISPRM [3]. Considering the results of the simple regression analyses, we think that the intramuscular adipose tissue can explain the muscle mass of the male and female older inpatients by approximately 55% and 46%, respectively.

The current study has two limitations. First, echo intensity has been reported to display not only intramuscular adipose tissue but also fibrosis tissue [37]. However, the results of muscle biopsy indicate that echo intensity is more strongly related to intramuscular adipose tissue than to fibrosis tissue [38]. In addition, the validity of the quadriceps echo intensity measurement for the intramuscular adipose tissue has also been indicated in a recent study using magnetic resonance imaging [25]. Second, we were not able to assess muscle function such as knee extension strength. Therefore, the relationship between quadriceps thickness and muscle strength in older inpatients is unclear. However, previous studies [5, 6, 39] reported that there are positive relationships between quadriceps thickness and knee extension strength in healthy older persons and non-ambulatory community-dwelling older persons and chronic stroke survivors.

## Conclusions

Our results show that there is a negative and significant association between muscle mass and fraction of intramuscular adipose tissue in older inpatients. Muscle quality of the quadriceps in older inpatients may be estimated to some extent by the muscle mass.

## Supporting information

S1 Data (XLSX)
